# Relevance of Phosphorylation and Truncation of Tau to the Etiopathogenesis of Alzheimer’s Disease

**DOI:** 10.3389/fnagi.2018.00027

**Published:** 2018-02-06

**Authors:** Yan Zhou, Jianhua Shi, Dandan Chu, Wen Hu, Zongyu Guan, Cheng-Xin Gong, Khalid Iqbal, Fei Liu

**Affiliations:** ^1^Key Laboratory of Neuroregeneration of Jiangsu and Ministry of Education of China, Co-innovation Center of Neuroregeneration, Nantong University, Nantong, China; ^2^Department of Neurochemistry, Inge Grundke-Iqbal Research Floor, New York State Institute for Basic Research in Developmental Disabilities, New York, NY, United States; ^3^Department of Biochemistry and Molecular Biology, School of Medicine, Nantong University, Nantong, China

**Keywords:** Alzheimer’s disease, tau, hyperphosphorylation, truncation, tau pathogenesis

## Abstract

Microtubule (MT) associated protein tau is abnormally hyperphosphorylated and aggregated into paired helical filaments (PHFs), which manifest as neurofibrillary tangles (NFTs) in the brains of individuals with Alzheimer’s disease (AD) and related tauopathies. Hyperphosphorylation and truncation of tau have been linked to the progression of the disease. However, the nature of phosphorylation and truncation of tau in AD brain are not very clear. In the present study we investigated the association of phosphorylation and truncation with high-molecular weight oligomers of tau (HMW-tau) in post-mortem AD brain by western blots. We found that tau from AD brain appears as a smear from low molecular weight (LMW) to HMW tau species in western blots developed with pan-tau antibodies. Similar level of LMW-tau was found in AD and control brains, whereas HMW-tau was found in AD brain only. HMW-tau was hyperphosphorylated at multiple sites and not unphosphorylated at Ser46 or Ser198/199/202. HMW-tau was weakly labeled by tau antibodies 43D against a.a. 6–18 and HT7 against a.a. 159–163 of tau, whereas, the C-terminal antibodies, tau46 and tau46.1, strongly labeled HMW-tau. The ratio of HMW-tau/LMW-tau detected by tau antibodies increased as the epitope of the tau antibodies ranges from N-terminal to C-terminal. The level of tau truncated at Asp421 was increased in AD brain, but was poorly associated with the HMW-tau. These findings suggest that tau pathogenesis involves both hyperphosphorylation and dominantly N-terminal truncation of tau in AD.

## Introduction

Alzheimer’s disease (AD) is characterized by extracellular deposits of β-amyloid plaques (Glenner et al., 1984) and intracellular neurofibrillary tangles (NFTs) consisting of abnormally hyperphosphorylated aggregates of the microtubule (MT) associated protein tau (Grundke-Iqbal et al., 1986a,b). The number of NFTs in the brain correlates to the severity of dementia symptoms in AD patients (Alafuzoff et al., 1987; Arriagada et al., 1992; Riley et al., 2002). Tau is a cytosolic phosphoprotein, the major function of which is to stimulate and stabilize MT assembly from tubulin subunits. Normal phosphorylation of tau controls its function in the regulation of MT dynamic, which is involved in neuronal polarity, axonal growth and axonal transportation. In AD brain, tau is abnormally hyperphosphorylated (Ksiezak-Reding et al., 1992; Köpke et al., 1993), which leads to the loss of biological activity, the gain of toxic activity, and the aggregation into paired helical filaments (PHFs; Iqbal et al., 1986; Alonso et al., 1994, 1996, 2001; Lucas et al., 2001; Fath et al., 2002; Jackson et al., 2002; Pérez et al., 2002).

In addition to phosphorylation, tau is post-translationally modified by ubiquitination (Mori et al., 1987; Perry et al., 1987), SUMOylation (Dorval and Fraser, 2006; Luo et al., 2014), glycation (Ledesma et al., 1994; Yan et al., 1994), acetylation (Min et al., 2010; Cohen et al., 2011), glycosylation (Wang et al., 1996b), O-GlcNAcylation (Arnold et al., 1996; Liu et al., 2004), nitration (Horiguchi et al., 2003), and truncation (Novak et al., 1993). Among them, truncation of tau has been shown to promote tau aggregation (Kovacech and Novak, 2010; Wang et al., 2010).

As a natively unfolded/poorly-folded protein, tau is very sensitive to protease digestion. Numerous studies have shown that tau is a substrate for calpain (Johnson et al., 1989), caspase (Gamblin et al., 2003), thrombin (Arai et al., 2005), cathepsin (Bednarski and Lynch, 1996), Puromycin-specific aminopeptidase (PSA; Karsten et al., 2006), and asparagine endopeptidase (Zhang et al., 2014) *in vitro* and *in vivo*. These studies have shown that tau truncation plays an important role in both tau aggregation and neurodegeneration (Kovacech and Novak, 2010). In AD brain, several specific truncations of tau have been identified (Wang et al., 2010). Truncation of tau at Asp421 (D421) and Glu391 (E391) has been shown to make tau prone to aggregation (Kovacech and Novak, 2010). However, the truncation of tau in the oligomeric forms in AD brain has not been well characterized.

Tau aggregated into oligomers and PHF in AD brain, which is visualized as a smear in western blots (Grundke-Iqbal et al., 1986a,b; Lee et al., 1991; Köpke et al., 1993). In the present study we investigated the phosphorylation and truncation of aggregated tau in post-mortem AD brain tissue by western blots. We found that high molecular weight tau oligomers (HMW-tau) from AD brain were hyperphosphorylated at multiple sites and truncated dominantly at the N-terminus.

## Materials and Methods

### Human Brain Tissue

Frozen frontal cortices from autopsied and histopathologically confirmed AD and age-matched normal human brains (Table 1) were obtained without identification of donors from the Sun Health Research Institute Donation Program (Sun City, AZ, USA). Brain samples were stored at −80°C until used. The use of autopsied frozen human brain tissue was in accordance with the National Institutes of Health guidelines and was exempted by the Institutional Review Board (IRB) of New York State Institute for Basic Research in Developmental Disabilities because “the research does not involve intervention or interaction with the individuals” nor “is the information individually identifiable”.

**Table 1:** Human brain tissue of Alzheimer’s disease (AD) and control (Con) cases used in this study.

Case	Age at death (Year)	Gender		PMI^a^(h)	Braak stage^b^	Tangle score^c^
		F	M			
AD (*n* = 17)	80.59 ± 6.70	10	7	2.52 ± 0.65	5.65 ± 0.49	13.57 ± 2.05
Con (*n* = 17)	82.59 ± 5.36	10	7	2.59 ± 0.53	2.18 ± 0.88	3.14 ± 1.89
AD (*n* = 10)^d^	76.60 ± 3.60	4	6	2.57 ± 0.71	5.70 ± 0.48	14.27 ± 1.40
Con (*n* = 10)^d^	79.80 ± 4.94	5	5	2.38 ± 0.55	1.70 ± 0.67	2.43 ± 1.86

*^a^PMI, postmortem interval; ^b^Neurofibrillary pathology was staged according to Braak and Braak (1991); ^c^Tangle score was a density estimate and was designated as none, sparse, moderate or frequent (0, 1, 2 or 3 for statistics), as defined according to CERAD Alzheimer’s disease criteria (Mirra et al., 1991). Five areas (frontal, temporal, parietal, hippocampal and entorhinal) were examined, and the scores were combined for a maximum of 15. ^d^Cases used in Figure 4*.

### Western Blots

The brain tissue was homogenized in cold buffer consisting of 50 mM Tris-HCl, pH 7.4, 8.5% sucrose, 2.0 mM EDTA, 10 mM β-mercaptoethanol, 1.0 mM orthovanadate, 50 mM NaF, 1.0 mM 4-(2-aminoethyl) benzenesulfonyl fluoride hydrochloride (AEBSF), and 10 μg/ml each of aprotinin, leupeptin, and pepstatin and stored at −80°C for western blots analysis. Brain homogenates were diluted in 2× Laemmli SDS sample buffer at 1:1 ratio, followed by boiling for 5 min. Samples were subjected to 7.5% SDS-PAGE and electrically blotted onto polyvinylidene fluoride membrane (Millipore) in transfer buffer (25 mM Tris-Glycine, pH 8.8, 20% methanol) at 0.5 ampere for 2 h. The membrane was subsequently blocked with 5% fat-free milk-TBS for 30 min, incubated with primary antibodies (Table 2) in TBS overnight, washed with TBST (TBS with 0.1% Tween20), incubated with HRP-conjugated secondary antibody for 2 h at RT, washed with TBST, and incubated with the ECL western Blotting Substrate (Thermo scientific) and exposed to HyBlot CL^®^ autoradiography film (Denville Scientific, Inc., Holliston, MA, USA). Specific immunostaining was quantified by using the Multi Gauge software V3.0 from Fuji Film.

**Table 2:** Primary antibodies employed in this study.

Antibody	Type	Species	Specificity	Reference/Source (catalog/lot number)
Anti-pT181-tau	Poly-	R	p-tau (T181)	Invitrogen
Anti-pS199-tau	Poly-	R	p-tau (S199)	Invitrogen (44734G)
AT8	Mono-	M	p-tau (S202/T205)	Thermo Scientific (MN1020)
Anti-pT205-tau	Poly-	R	p-tau (T205)	Invitrogen (44738G)
Anti-pT212-tau	Poly-	R	p-tau (T212)	Invitrogen (44740G)
Anti-pS214-tau	Poly-	R	p-tau (S214)	Invitrogen (44742G)
Anti-pT217-tau	Poly-	R	p-tau (T217)	Invitrogen (44744)
12E8	Mono-	M	p-tau (S262/356)	Dr. D. Schenk
Anti-pS396-tau	Poly-	R	p-tau (S396)	Invitrogen (44752G)
Anti-pS404-tau	Poly-	R	p-tau (S404)	Invitrogen (44–758G)
R145d	Poly-	R	p-tau (S422)	Pei et al. (1998)
92e	Poly-	R	Pan-tau	Pei et al. (1998)
RD3	Mono-	M	3R-tau	Millipore (05–803/JBC1863429)
RD4	Mono-	M	4R-tau	Millipore (05–804/2073108)
Tau-1	Mono-	M	Up-tau (S199/202)	Binder et al. (1985)
QCB23070	Poly-	R	Up-tau (S46)	Gong et al. (1994)
43D	Mono-	M	Pan-tau (a.a. 6–18)	Liu et al. (2009)
HT7	Mono-	M	Pan-tau (a.a. 159–163)	Thermo Scientific (MN10000)
Tau 46.1	Mono-	M	Pan-tau (a.a. 428–441)	Millipore (05–838-MI)
Tau 46	Mono-	M	Pan-tau (a.a. 404–421)	Invitrogen (13–6400)
Tau C3	Mono-	M	Truncated tau at D421	Invitrogen (AHB0061)
Anti-GAPDH	Poly-	R	GAPDH	Santa-Cruz (sc-25778)

*Abbreviations: Mono-, monoclonal; p-, phosphorylated; Up-, unphosphorylated; Poly-, polyclonal; M, Mouse; R, Rabbit*.

### Statistical Analysis

Data points were compared by unpaired two-tailed Student’s *t*-test (for data with normal distribution) or Mann-Whitney test (for data with non-normal distribution) and one-way analysis of variance (ANOVA) followed by Tukey’s multiple comparisons test. The data are presented as the mean ± SD. To reveal the association between high molecular weight aggregated tau and position of the epitope of the antibodies, Spearman correlation analysis was performed. *p* < 0.05 was considered statistically significant.

## Results

### HMW-tau Is Present in AD Brain Homogenates

**Figure 1:**
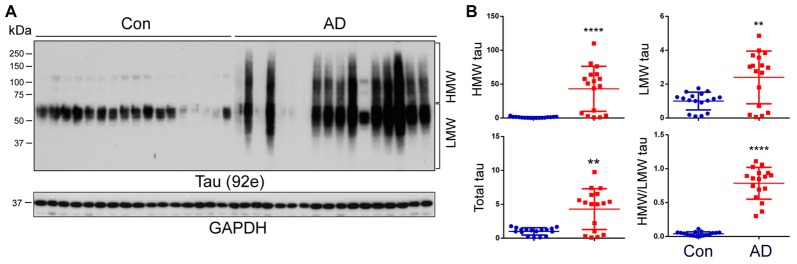
High-molecular weight tau (HMW-tau) is selectively present in Alzheimer’s disease (AD) brain. **(A)** Frontal cortical homogenates from 17 control and 17 AD cases were analyzed by western blots developed with a pan-tau antibody, 92e. **(B)** Blots were analyzed by densitometry. The levels of total tau, HMW-tau, low molecular tau (LMW-tau), and ratio of the HMW-tau/LMW-tau are presented as scattered dots with mean ± SD. **p < 0.01; ****p < 0.0001.

The aggregated tau forms oligomers and PHFs in AD brain, which are seen as a smear on western blots (Grundke-Iqbal et al., 1986a,b; Lee et al., 1991). To learn the nature of tau in AD and control brains, frontal cortical homogenates from 17 controls and 17 AD cases from two cohorts (Table 1) were analyzed by western blots developed with a pan-tau antibody, 92e. We observed several bands of tau from 50 kDa to 65 kDa immuno-recognized by 92e in control brains (Figure 1A). Tau in AD brain homogenates appeared as a smear (Figure 1A). We quantified the level of total tau, HMW-tau (65 kDa upwards) and low molecular weight (LMW-tau; 65 kDa downwards), as indicated in Figure 1A, by densitometry. We found that the total level of tau was ~5-fold increase in AD brains (Figures 1A,B). The HMW-tau, which is considered as aggregated tau, was only seen in AD brain (Figure 1B). Lower molecular weight tau (LMW-tau) was increased by about 2.5-fold in AD brain (Figure 1B). The ratio of HMW-tau/LMW-tau was 0.78 in AD and ~ 0.04 in control cases. These results suggest that AD brain is characterized by a smeared aggregation of tau.

### HMW-tau Is Abnormally Hyperphosphorylated at Multiple Sites in AD Brain

**Figure 2:**
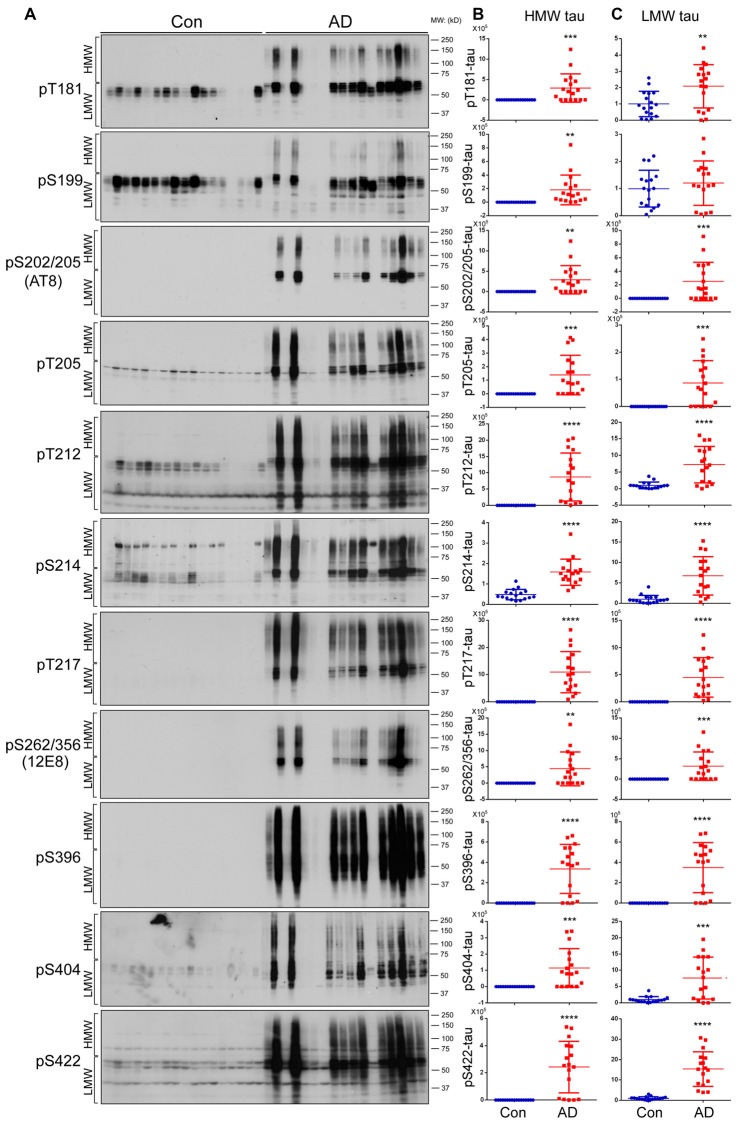
Both HMW-tau and LMW-tau are selectively hyperphosphorylated in AD brain. **(A)** AD and control human brain homogenates were analyzed by western blots developed with the indicated site-specific and phosphorylation dependent anti-tau antibodies. **(B,C)** Blots were analyzed by densitometry. The levels of hyperphosphorylated HMW-tau **(B)** and LMW-tau **(C)** are shown as scattered dots with mean ± SD. **p < 0.01; ***p < 0.001; ****p < 0.0001.

Tau is hyperphosphorylated in AD brain at multiple sites (Köpke et al., 1993; Liu et al., 2009). To determine association of phosphorylation with HMW-tau, we analyzed tau phosphorylation in AD and control brain homogenates by western blots developed with several site-specific and phosphorylation dependent tau antibodies (Table 2). We found that phosphorylation of HMW-tau in control brains was barely detectable (Figure 2A). In contrast in AD brains, HMW-tau was phosphorylated at all phosphorylation sites studied, which include Ser181, Ser199, Ser202/Thr205, Thr205, Thr212, Ser214, Thr217, Ser262/356, Ser396, Ser404 and Ser422 (Figures 2A,B). Compared with control brains, phosphorylation level of LMW-tau was increased at all studied phosphorylation sites, except Ser199, in AD brains (Figures 2A,C). These results suggest that hyperphosphorylated tau is strongly associated with HMW-tau.

**Figure 3:**
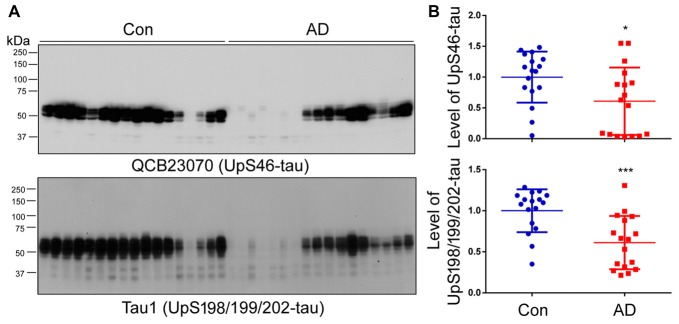
Tau unphosphorylated at Ser46 and Ser198/199/202 is not present in the HMW-tau species. **(A)** The levels of tau unphosphorylated at Ser46 and Ser198/199/202 in human brain homogenates were determined by western blots. **(B)** Blots were analyzed by densitometry. Quantification is presented as scattered dots with mean ± SD. *p < 0.05; ***p < 0.001.

To investigate whether non-hyperphosphorylated tau is also present in the HMW-tau smears, the western blots of above brain homogenates were developed with QCB23070 against tau unphosphorylated at Ser46 and Tau1 against tau unphosphorylated at Ser198/199/202, respectively. We found that the level of tau recognized by either QCB23070 or Tau-1 was decreased in AD brain (Figure 3). Furthermore, HMW-tau in AD brain was recognized by neither QCB23070 nor Tau-1, suggesting that HMW-tau is phosphorylated at both Ser46 and Ser198/199/202. This finding confirms the involvement of hyperphosphorylation of tau in AD pathogenesis.

### N-terminal Truncation of Tau Is More Associated than Its C-terminal Truncation with HMW-tau in AD

**Figure 4:**
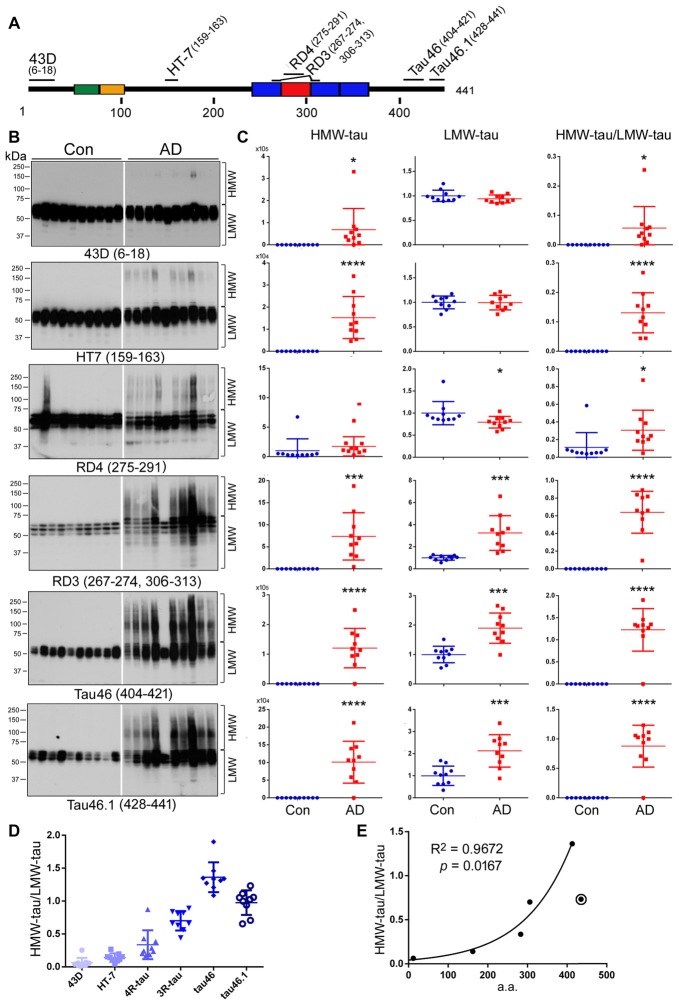
HWM-tau is mainly truncated at the N-terminus. **(A)** Schematic diagram of the epitopes of tau antibodies used in the present study. **(B,C)** Western blots of brain homogenates from 10 control and 10 AD cases developed with six tau antibodies indicated under each blot and analyzed by densitometry. The levels of HMW-tau and LMW-tau and the ratio of HMW-tau/LMW-tau are presented as scattered dots with mean ± SD. *p < 0.05, ***p < 0.001, ****p < 0.0001. **(D)** The ratios of HMW-tau/LMW-tau detected by individual tau antibodies were present as scattered dots with mean ± SD. The levels detected by all antibodies are significantly different, except between by HT-7 and by RD4 by repeated measures analysis of variance (ANOVA) followed by Tukey’s multiple comparisons test. **(E)** The mean ratio of HWM-tau/LMW-tau detected by each antibody was plotted against the medium a.a. of corresponding epitope. Spearman correlation analysis was performed by including antibodies 43D, HT7, RD4, RD3 and Tau46, but not Tau46.1, which is marked with a circle.

Several truncations of tau have been identified in AD brain, especially in NFTs (Novak et al., 1991; Basurto-Islas et al., 2008). To learn the N- and C-terminal truncations of HMW-tau, we analyzed tau in the above 10 AD and 10 control cases (see Table 1) by western blots developed with phosphorylation-independent monoclonal antibodies against different regions of the tau molecule, including 43D (a.a. 6–18), HT7 (a.a. 159–163), RD4 (a.a. 275–291), RD3 (a.a. 267–274 and 306–313), Tau46 (a.a. 404–421) and Tau46.1 (a.a. 428–441; Figure 4A). Consistent with above studies, no HMW-tau was observed in control brain by any of the antibodies used in the present study, except Con 2 by RD4 (Figure 4B). Level of LMW-tau in AD brain was similar to that in control brain detected with 43D and HT7, ~2-fold increased with RD3, Tau46 and Tau46.1, and slightly decreased with RD4 (Figures 4B,C). These data suggest that the levels of total LMW-tau and LMW-3R-tau are increased and LMW-4R-tau is decreased in AD brain. HMW-tau was present in AD brains detected with all the antibodies (Figures 4B,C). HMW-tau immunoreacted weakly with N-terminal antibody 43D and HT7, and strongly with C-terminal antibodies Tau46 and Tau46.1 (Figure 4B). The level of HMW-tau was gradually increased with antibodies epitopes range from N-terminal to C-terminal (Figure 4B). The ratio of HMW/LMW was dramatically increased in AD brain compared with control brains (Figure 4C). These results suggest that the N-terminus may be truncated in the HMW-tau.

Subsequently, we compared the ratio of HMW-tau/LMW-tau in AD brains detected by these six antibodies against different tau epitopes. We found that the ratios of HMW-tau/LMW were significantly different, except between HT-7 and RD4. The ratio of HMW-tau/LMW-tau showed a significant increase as the epitopes of tau antibodies range from N-terminal to C terminal (Figure 4D), suggesting that truncation of tau at the N-terminus increases its aggregation. Furthermore, the ratio of HMW-tau/LMW-tau with Tau46.1 (a.a. 428–441) was decreased as compared with that detected by Tau46, suggesting that truncation of the last 14 amino acids of tau may also increase in the HMW-tau.

To investigate the association of tau truncation with ratio of HMW-tau/LMW-tau, we plotted the ratio against the epitope of each antibody, for which medium a.a. of the epitope of each antibody was employed (Figure 4E). We observed a strong correlation between the ratio of HMW-tau/LMW-tau and the epitope location of tau antibodies, including 43D, HT7, RD4, RD3 and Tau46 (Figure 4E). These findings suggest that C-terminal part of tau is associated with HMW-tau more than the N-terminal portion, but proximal C-terminal portion of tau may suppress the aggregation.

### D421 Truncation Is Increased in AD Brain, but Not Associated with HMW-tau

**Figure 5:**
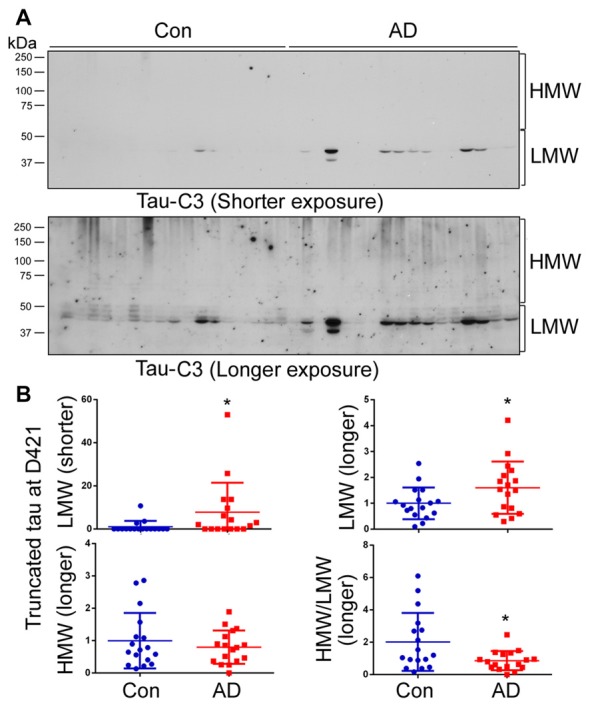
Tau truncated at D421 does not associate with HMW-tau in AD brains. **(A)** Level of tau_D421_ was determined by western blots developed with Tau-C3 antibody using short or long exposures. **(B)** Blots were quantified by densitometry. The levels of HMW-tau_D421_ and LMW-tau_D421_ and the ratio of HMW-tau_D421_/LMW-tau_D421_ are shown as scattered dots with mean ± SD. *p < 0.05.

Truncation of tau at Aps421 (tau_D421_) has been identified in AD brain, especially in NFTs, and it is believed that this truncation may enhance the aggregation of tau into filaments (Abraha et al., 2000; Gamblin et al., 2003). To determine the relevance of D421 truncation in tau aggregation in AD brain, we analyzed the brain homogenates by western blots developed with Tau-C3, which specifically recognizes tau_D421_. By analyzing blots developed for short and long exposure time (Figure 5A), we found only a weak signal and no difference in the level of tau_D421_ in HMW-tau (Figure 5B); a ~2-fold increase was, however, seen in LMW-tau in AD (Figure 5B). The ratio of HMW-tau_D421_/LMW-tau_D421_ was decreased in AD brain (Figure 5B). These data suggest that C-terminal D421 truncation is probably not a significant player in HMW-tau in AD.

## Discussion

Conversion of monomeric tau to oligomeric and filamentous aggregates is apparently central to tau pathogenesis in AD and related tauopathies (Iqbal et al., 2016). Tau in AD, especially in NFTs is hyperphosphorylated and truncated. In the present study, by analyzing post-mortem brain tissues, we determined the relevance of hyperphosphorylation and truncation of tau in HMW-tau aggregates in AD brain. We found HMW-tau only in AD brain, but not in control brain. The HMW-tau was hyperphosphorylated at Thr181, Ser199, Ser202, Thr205, Thr212, Ser214, Thr217, Ser262, Ser396, Ser404 and Ser422. Tau unphosphorylated at Ser46 or Ser198/199/202 was not found in the HMW-tau aggregates. HMW-tau lacked the extreme N-terminal portion of tau suggesting that N-terminal truncated tau should be more relevant than the C-terminal truncated tau in HMW-tau. Tau truncated at D421, tau_D421_, was increased in AD brain, but not significantly associated with the HMW-tau as detected by western blots. The level of tau with extreme C-terminus, a.a. 428–441 was reduced in HMW-tau. These findings suggest that hyperphosphorylated and N-terminal truncated and not C-terminal truncated tau is apparently significant constituent of HMW-tau.

In AD brain, tau pathology starts from the entorhinal cortex, spreads to the hippocampus and frontal and temporal cortices, and finally to all isocortex areas, but the cerebellum is spared from tau lesions. Six stages of disease propagation are distinguished with respect to the location of the tangle-bearing neurons and the severity of changes by Braak. Braak stages I and II are used when NFT involvement is confined mainly to the transentorhinal region of the brain, stages III and IV when there is also involvement of limbic regions such as the hippocampus, and V and VI when there is extensive neocortical involvement (Braak and Braak, 1991). In the present study, we analyzed tau in frontal cortex by western blots. All control cases used for this study are Braak stage I–IV, at which frontal cortex may not develop tau lesion. However, all AD cases are Braak Stage V–VI, in which tau pathology spreads to all isocortical areas, including frontal cortex. We found a great level of HMW-tau and truncation of tau species in AD brain detected by various anti-tau antibodies. Furthermore, we speculate a higher level of HMW-tau and truncated tau in entorhinal cortex and the hippocampus, brain regions in which more advanced tau pathology would be expected.

As a phospho-protein, the biological activity of tau is regulated by its degree of phosphorylation. Tau441, the longest tau isoform in the human brain, has 80 Ser/Thr and 5 Tyr potential phosphorylation sites. Normal human brain tau contains 2–3 moles phosphates/mole of the protein, while the abnormally hyperphosphorylated tau from AD brain has a phosphorylation stoichiometry of 9–10 (Köpke et al., 1993). To date, more than 40 phosphorylation sites of tau from AD brain have been identified (Wang and Liu, 2008). Compared with the age- and post-mortem match controls, the level of tau phosphorylation at multiple sites is increased significantly in AD brain (Liu et al., 2009). In the present study, we found that the level of phosphorylation of tau at all phosphorylation sites examined is dramatically increased and is greatly associated with HMW-tau in AD brain, suggesting a great relevance of the hyperphosphorylation with the aggregation of tau.

Hyperphosphorylated tau from AD brain (AD p-tau), the major protein subunit of PHF, is able to self-assemble into PHF and is unable to promote the assembly of MTs *in vitro* (Alonso et al., 1994). Dephosphorylation of AD p-tau by protein phosphatases restores its biological activity to promote assembly of MTs and inhibits its self-polymerization into PHF/straight filaments (SF; Wang et al., 1996a; Wang J. Z. et al., 2007). Phosphorylation of recombinant tau by PKA or Dyrk1A suppresses its capability of promoting MT assembly (Liu et al., 2007) and phosphorylation by the kinases in rat brain extract induces the self-assembly of tau into tangles of PHF and SF (Alonso et al., 2001). Furthermore, AD p-tau sequestrates normal tau into tangles of filaments (Alonso et al., 1996), indicating its prion-like property. Dephosphorylation of AD P-tau with alkaline phosphatase abolishes its ability to aggregate with normal tau and prevents tangle formation (Alonso et al., 1996). Furthermore, propagation of AD p-tau *in vivo* is altered by dephosphorylation with PP2A, suggesting hyperphosphorylation determines its prion-like spreading (Hu et al., 2016).

Tau protein is natively unfolded or intrinsically disordered and has little tendency for aggregation (Mukrasch et al., 2009). However, the tau molecule shows a preference for changing its global conformations to form a paperclip-like conformation by folding the N- and C-terminal portions back on the MT binding repeats (Jeganathan et al., 2006), which might protect tau from aggregation. Tau fragments truncated at both N- and C-termini that contain MT binding repeats have a higher tendency for aggregation (Wang Y. P. et al., 2007), probably resulting from the disruption of the paperclip structure. Thus, both the N- and C-termini appear to have an inhibitory effect on the aggregation potential of tau. The N-terminal fragments of tau inhibit tau441 polymerization by interacting with a specific C-terminal sequences and stabilizing a soluble conformation of tau (Horowitz et al., 2006). In the present study, we found that in the HWM-tau from frontal cortex of AD was barely immunorecognized by 43D, suggesting that the HMW-tau lacks the N-terminus and that the N-terminal truncation of tau may promote tau aggregation to form NFTs *in vivo*. Interestingly, we recently found that 43D effectively inhibits tau pathology in 3XTg-AD mice (Dai et al., 2017). Since normal tau can be sequestered by hyperphosphorylated tau from AD brain (Alonso et al., 1996), we speculate that 43D suppresses tau pathology by acting on normal tau which is-associated with pathological tau.

Tau is a substrate for various proteases (Wang et al., 2010). It can be cleaved by caspase 6 at Asp13 and Asp402, by caspase 3 at Asp25 and ASP421, by chymotrypsin at Tyr197, by an unknown thrombin-like cytosolic protease at Lys257, by asparaginyl endopeptidase at Asn255 and Asn368, and by calpain at Lys44 and Arg230 (Wang et al., 2010). Glu391 is cleaved by an unknown protease (Wang et al., 2010). PSA proteolyzes residues stepwise from the N-terminus of tau (Wang et al., 2010; Wang and Mandelkow, 2016). Among all truncations of tau, truncations at Glu (E391) and Asp (D421) have been mostly reported in AD brain. Both truncations were reported to make tau proteins more prone to aggregation than the full-length tau (Abraha et al., 2000; Berry et al., 2003; Gamblin et al., 2003; Yin and Kuret, 2006). Tau_E391_ not only greatly increases the rate of *in vitro* filament formation, but also increases the amount of the tau protein assembled into fibrillar structures (Abraha et al., 2000). PHFs isolated from AD brain and treated with pronase (to remove the fuzzy coat) contain tau fragment tau_151–391_ (Wischik et al., 1988), which is prone to aggregation. Rats transgenic for tau_151–391_ develop neurofibrillary pathology and also display hyperphosphorylation and production of HMW-tau species (Zilka et al., 2006). However, in the present study, we observed a great level of C-terminal portion of tau in HMW-tau detected by antibodies Tau46 and Tau46.1, suggesting that majority of tau in AD brain is not truncated at the C-terminal. It is reported that psedophosphorylation of tau at Ser422 suppresses D421 or E391 truncation (Guillozet-Bongaarts et al., 2006). In AD brain, tau is hyperphosphorylated at Ser396, Ser404 and Ser422, which may inhibit these two C-terminal truncations. However, tau_D421_ was increased and the level of tau with intact C-terminal end as detected by tau46.1 was significantly lower in HMW-tau than that of tau recognized by Tau46, suggesting that this C-terminal truncation may be involved in tau aggregation.

In conclusion, in the present study we found that HMW-tau are hyperphosphorylated at multiple sites, and unphosphorylated tau, such as at Ser46 and at Ser198/199/202, is absent in these aggregates. The N-terminal truncated tau is associated with HMW-tau more than the C-terminal truncated tau. These findings provide a novel insight into the association of the hyperphosphorylation and truncation of tau with tau aggregation into oligomers in AD.

## Author Contributions

FL, WH and KI: conception of the research. YZ, JS, DC, ZG and FL: performing experiments. YZ, WH and FL: analyses and interpretation of results. WH and FL: drafting of the manuscript. WH, C-XG and KI: critical revision of the manuscript.

## Conflict of Interest Statement

The authors declare that the research was conducted in the absence of any commercial or financial relationships that could be construed as a potential conflict of interest.

## References

[B1] Abraha A. Ghoshal N. Gamblin T. C. Cryns V. Berry R. W. Kuret J.. (2000). C-terminal inhibition of tau assembly *in vitro* and in Alzheimer’s disease. J. Cell Sci. 113, 3737–3745. 11034902 10.1242/jcs.113.21.3737

[B2] Alafuzoff I. Iqbal K. Friden H. Adolfsson R. Winblad B. (1987). Histopathological criteria for progressive dementia disorders: clinical-pathological correlation and classification by multivariate data analysis. Acta Neuropathol. 74, 209–225. 10.1007/bf00688184 3673513

[B4] Alonso A. C. Grundke-Iqbal I. Iqbal K. (1996). Alzheimer’s disease hyperphosphorylated tau sequesters normal tau into tangles of filaments and disassembles microtubules. Nat. Med. 2, 783–787. 10.1038/nm0796-783 8673924

[B5] Alonso A. C. Zaidi T. Grundke-Iqbal I. Iqbal K. (1994). Role of abnormally phosphorylated tau in the breakdown of microtubules in Alzheimer disease. Proc. Natl. Acad. Sci. U S A 91, 5562–5566. 10.1073/pnas.91.12.5562 8202528 PMC44036

[B3] Alonso A. Zaidi T. Novak M. Grundke-Iqbal I. Iqbal K. (2001). Hyperphosphorylation induces self-assembly of tau into tangles of paired helical filaments/straight filaments. Proc. Natl. Acad. Sci. U S A 98, 6923–6928. 10.1073/pnas.121119298 11381127 PMC34454

[B6] Arai T. Guo J. P. McGeer P. L. (2005). Proteolysis of non-phosphorylated and phosphorylated tau by thrombin. J. Biol. Chem. 280, 5145–5153. 10.1074/jbc.M409234200 15542598

[B7] Arnold C. S. Johnson G. V. Cole R. N. Dong D. L. Lee M. Hart G. W. (1996). The microtubule-associated protein tau is extensively modified with O-linked N-acetylglucosamine. J. Biol. Chem. 271, 28741–28744. 10.1074/jbc.271.46.28741 8910513

[B8] Arriagada P. V. Growdon J. H. Hedley-Whyte E. T. Hyman B. T. (1992). Neurofibrillary tangles but not senile plaques parallel duration and severity of Alzheimer’s disease. Neurology 42, 631–639. 10.1212/wnl.42.3.631 1549228

[B9] Basurto-Islas G. Luna-Muñoz J. Guillozet-Bongaarts A. L. Binder L. I. Mena R. García-Sierra F. (2008). Accumulation of aspartic acid421- and glutamic acid391-cleaved tau in neurofibrillary tangles correlates with progression in Alzheimer disease. J. Neuropathol. Exp. Neurol. 67, 470–483. 10.1097/NEN.0b013e31817275c7 18431250 PMC4699801

[B10] Bednarski E. Lynch G. (1996). Cytosolic proteolysis of tau by cathepsin D in hippocampus following suppression of cathepsins B and L. J. Neurochem. 67, 1846–1855. 10.1046/j.1471-4159.1996.67051846.x 8863489

[B11] Berry R. W. Abraha A. Lagalwar S. LaPointe N. Gamblin T. C. Cryns V. L.. (2003). Inhibition of tau polymerization by its carboxy-terminal caspase cleavage fragment. Biochemistry 42, 8325–8331. 10.1021/bi027348m 12846581

[B80] Binder L. I. Frankfurter A. Rebhun L. I. (1985). The distribution of tau in the mammalian central nervous system. J. Cell Biol. 101,1371–1378. 10.1083/jcb.101.4.1371 3930508 PMC2113928

[B12] Braak H. Braak E. (1991). Neuropathological stageing of Alzheimer-related changes. Acta Neuropathol. 82, 239–259. 10.1007/bf00308809 1759558

[B14] Cohen T. J. Guo J. L. Hurtado D. E. Kwong L. K. Mills I. P. Trojanowski J. Q.. (2011). The acetylation of tau inhibits its function and promotes pathological tau aggregation. Nat. Commun. 2:252. 10.1038/ncomms1255 21427723 PMC3120096

[B15] Dai C. L. Tung Y. C. Liu F. Gong C. X. Iqbal K. (2017). Tau passive immunization inhibits not only tau but also Aβ pathology. Alzheimers Res. Ther. 9:1. 10.1186/s13195-016-0227-5 28073379 PMC5225540

[B16] Dorval V. Fraser P. E. (2006). Small ubiquitin-like modifier (SUMO) modification of natively unfolded proteins tau and α-synuclein. J. Biol. Chem. 281, 9919–9924. 10.1074/jbc.M510127200 16464864

[B17] Fath T. Eidenmuller J. Brandt R. (2002). Tau-mediated cytotoxicity in a pseudohyperphosphorylation model of Alzheimer’s disease. J. Neurosci. 22, 9733–9741. 12427828 10.1523/JNEUROSCI.22-22-09733.2002 PMC6757822

[B18] Gamblin T. C. Chen F. Zambrano A. Abraha A. Lagalwar S. Guillozet A. L.. (2003). Caspase cleavage of tau: linking amyloid and neurofibrillary tangles in Alzheimer’s disease. Proc. Natl. Acad. Sci. U S A 100, 10032–10037. 10.1073/pnas.1630428100 12888622 PMC187753

[B19] Glenner G. G. Wong C. W. Quaranta V. Eanes E. D. (1984). The amyloid deposits in Alzheimer’s disease: their nature and pathogenesis. Appl. Pathol. 2, 357–369. 6242724

[B20] Gong C. X. Grundke-Iqbal I. Iqbal K. (1994). Dephosphorylation of Alzheimer’s disease abnormally phosphorylated tau by protein phosphatase-2A. Neuroscience 61, 765–772. 10.1016/0306-4522(94)90400-6 7838376

[B21] Grundke-Iqbal I. Iqbal K. Quinlan M. Tung Y. C. Zaidi M. S. Wisniewski H. M. (1986a). Microtubule-associated protein tau. A component of Alzheimer paired helical filaments. J. Biol. Chem. 261, 6084–6089. 3084478

[B22] Grundke-Iqbal I. Iqbal K. Tung Y. C. Quinlan M. Wisniewski H. M. Binder L. I. (1986b). Abnormal phosphorylation of the microtubule-associated protein tau (tau) in Alzheimer cytoskeletal pathology. Proc. Natl. Acad. Sci. U S A 83, 4913–4917. 10.1073/pnas.83.13.4913 3088567 PMC323854

[B23] Guillozet-Bongaarts A. L. Cahill M. E. Cryns V. L. Reynolds M. R. Berry R. W. Binder L. I. (2006). Pseudophosphorylation of tau at serine 422 inhibits caspase cleavage: *in vitro* evidence and implications for tangle formation *in vivo*. J. Neurochem. 97, 1005–1014. 10.1111/j.1471-4159.2006.03784.x 16606369

[B24] Horiguchi T. Uryu K. Giasson B. I. Ischiropoulos H. LightFoot R. Bellmann C.. (2003). Nitration of tau protein is linked to neurodegeneration in tauopathies. Am. J. Pathol. 163, 1021–1031. 10.1016/s0002-9440(10)63462-1 12937143 PMC1868254

[B25] Horowitz P. M. LaPointe N. Guillozet-Bongaarts A. L. Berry R. W. Binder L. I. (2006). N-terminal fragments of tau inhibit full-length tau polymerization *in vitro*. Biochemistry 45, 12859–12866. 10.1021/bi061325g 17042504

[B26] Hu W. Zhang X. Tung Y. C. Xie S. Liu F. Iqbal K. (2016). Hyperphosphorylation determines both the spread and the morphology of tau pathology. Alzheimers Dement. 12, 1066–1077. 10.1016/j.jalz.2016.01.014 27133892

[B27] Iqbal K. Grundke-Iqbal I. Zaidi T. Merz P. A. Wen G. Y. Shaikh S. S.. (1986). Defective brain microtubule assembly in Alzheimer’s disease. Lancet 2, 421–426. 10.1016/s0140-6736(86)92134-3 2874414

[B28] Iqbal K. Liu F. Gong C. X. (2016). Tau and neurodegenerative disease: the story so far. Nat. Rev. Neurol. 12, 15–27. 10.1038/nrneurol.2015.225 26635213

[B29] Jackson G. R. Wiedau-Pazos M. Sang T. K. Wagle N. Brown C. A. Massachi S.. (2002). Human wild-type tau interacts with wingless pathway components and produces neurofibrillary pathology in Drosophila. Neuron 34, 509–519. 10.1016/s0896-6273(02)00706-7 12062036

[B30] Jeganathan S. von Bergen M. Brutlach H. Steinhoff H. J. Mandelkow E. (2006). Global hairpin folding of tau in solution. Biochemistry 45, 2283–2293. 10.1021/bi0521543 16475817

[B31] Johnson G. V. Jope R. S. Binder L. I. (1989). Proteolysis of tau by calpain. Biochem. Biophys. Res. Commun. 163, 1505–1511. 10.1016/0006-291x(89)91150-9 2551295

[B32] Karsten S. L. Sang T. K. Gehman L. T. Chatterjee S. Liu J. Lawless G. M.. (2006). A genomic screen for modifiers of tauopathy identifies puromycin-sensitive aminopeptidase as an inhibitor of tau-induced neurodegeneration. Neuron 51, 549–560. 10.1016/j.neuron.2006.07.019 16950154

[B34] Köpke E. Tung Y. C. Shaikh S. Alonso A. C. Iqbal K. Grundke-Iqbal I. (1993). Microtubule-associated protein tau. Abnormal phosphorylation of a non-paired helical filament pool in Alzheimer disease. J. Biol. Chem. 268, 24374–24384. 8226987

[B35] Kovacech B. Novak M. (2010). Tau truncation is a productive posttranslational modification of neurofibrillary degeneration in Alzheimer’s disease. Curr. Alzheimer Res. 7, 708–716. 10.2174/156720510793611556 20678071

[B36] Ksiezak-Reding H. Liu W. K. Yen S. H. (1992). Phosphate analysis and dephosphorylation of modified tau associated with paired helical filaments. Brain Res. 597, 209–219. 10.1016/0006-8993(92)91476-u 1472994

[B37] Ledesma M. D. Bonay P. Colaco C. Avila J. (1994). Analysis of microtubule-associated protein tau glycation in paired helical filaments. J. Biol. Chem. 269, 21614–21619. 8063802

[B38] Lee V. M. Balin B. J. Otvos L. Jr. Trojanowski J. Q. (1991). A68: a major subunit of paired helical filaments and derivatized forms of normal Tau. Science 251, 675–678. 10.1126/science.1899488 1899488

[B39] Liu F. Iqbal K. Grundke-Iqbal I. Hart G. W. Gong C. X. (2004). O-GlcNAcylation regulates phosphorylation of tau: a mechanism involved in Alzheimer’s disease. Proc. Natl. Acad. Sci. U S A 101, 10804–10809. 10.1073/pnas.0400348101 15249677 PMC490015

[B40] Liu F. Li B. Tung E. J. Grundke-Iqbal I. Iqbal K. Gong C. X. (2007). Site-specific effects of tau phosphorylation on its microtubule assembly activity and self-aggregation. Eur. J. Neurosci. 26, 3429–3436. 10.1111/j.1460-9568.2007.05955.x 18052981 PMC2262108

[B41] Liu F. Shi J. Tanimukai H. Gu J. Gu J. Grundke-Iqbal I.. (2009). Reduced O-GlcNAcylation links lower brain glucose metabolism and tau pathology in Alzheimer’s disease. Brain 132, 1820–1832. 10.1093/brain/awp099 19451179 PMC2702834

[B42] Lucas J. J. Hernández F. Gómez-Ramos P. Morán M. A. Hen R. Avila J. (2001). Decreased nuclear β-catenin, tau hyperphosphorylation and neurodegeneration in GSK-3β conditional transgenic mice. EMBO J. 20, 27–39. 10.1093/emboj/20.1.27 11226152 PMC140191

[B43] Luo H. B. Xia Y. Y. Shu X. J. Liu Z. C. Feng Y. Liu X. H.. (2014). SUMOylation at K340 inhibits tau degradation through deregulating its phosphorylation and ubiquitination. Proc. Natl. Acad. Sci. U S A 111, 16586–16591. 10.1073/pnas.1417548111 25378699 PMC4246270

[B44] Min S. W. Cho S. H. Zhou Y. Schroeder S. Haroutunian V. Seeley W. W.. (2010). Acetylation of tau inhibits its degradation and contributes to tauopathy. Neuron 67, 953–966. 10.1016/j.neuron.2010.08.044 20869593 PMC3035103

[B45] Mirra S. S. Heyman A. McKeel D. Sumi S. M. Crain B. J. Brownlee L. M.. (1991). The consortium to establish a registry for Alzheimer’s disease (CERAD). Part II. Standardization of the neuropathologic assessment of Alzheimer’s disease. Neurology 41, 479–486. 10.1212/WNL.41.4.479 2011243

[B46] Mori H. Kondo J. Ihara Y. (1987). Ubiquitin is a component of paired helical filaments in Alzheimer’s disease. Science 235, 1641–1644. 10.1126/science.3029875 3029875

[B47] Mukrasch M. D. Bibow S. Korukottu J. Jeganathan S. Biernat J. Griesinger C.. (2009). Structural polymorphism of 441-residue tau at single residue resolution. PLoS Biol. 7:e34. 10.1371/journal.pbio.1000034 19226187 PMC2642882

[B48] Novak M. Jakes R. Edwards P. C. Milstein C. Wischik C. M. (1991). Difference between the tau protein of Alzheimer paired helical filament core and normal tau revealed by epitope analysis of monoclonal antibodies 423 and 7.51. Proc. Natl. Acad. Sci. U S A 88, 5837–5841. 10.1073/pnas.88.13.5837 1712107 PMC51973

[B49] Novak M. Kabat J. Wischik C. M. (1993). Molecular characterization of the minimal protease resistant tau unit of the Alzheimer’s disease paired helical filament. EMBO J. 12, 365–370. 7679073 10.1002/j.1460-2075.1993.tb05665.x PMC413214

[B50] Pei J. J. Gong C. X. Iqbal K. Grundke-Iqbal I. Wu Q. L. Winblad B.. (1998). Subcellular distribution of protein phosphatases and abnormally phosphorylated tau in the temporal cortex from Alzheimer’s disease and control brains. J. Neural. Transm. 105, 69–83. 10.1007/s007020050039 9588762

[B51] Pérez M. Hernández F. Gómez-Ramos A. Smith M. Perry G. Avila J. (2002). Formation of aberrant phosphotau fibrillar polymers in neural cultured cells. Eur. J. Biochem. 269, 1484–1489. 10.1046/j.1432-1033.2002.02794.x 11874463

[B52] Perry G. Friedman R. Shaw G. Chau V. (1987). Ubiquitin is detected in neurofibrillary tangles and senile plaque neurites of Alzheimer disease brains. Proc. Natl. Acad. Sci. U S A 84, 3033–3036. 10.1073/pnas.84.9.3033 3033674 PMC304795

[B53] Riley K. P. Snowdon D. A. Markesbery W. R. (2002). Alzheimer’s neurofibrillary pathology and the spectrum of cognitive function: findings from the Nun Study. Ann. Neurol. 51, 567–577. 10.1002/ana.10161 12112102

[B60] Wang Y. P. Biernat J. Pickhardt M. Mandelkow E. Mandelkow E. M. (2007). Stepwise proteolysis liberates tau fragments that nucleate the Alzheimer-like aggregation of full-length tau in a neuronal cell model. Proc. Natl. Acad. Sci. U S A 104, 10252–10257. 10.1073/pnas.0703676104 17535890 PMC1891218

[B58] Wang Y. Garg S. Mandelkow E. M. Mandelkow E. (2010). Proteolytic processing of tau. Biochem. Soc. Trans. 38, 955–961. 10.1042/BST0380955 20658984

[B54] Wang J. Z. Grundke-Iqbal I. Iqbal K. (1996a). Restoration of biological activity of Alzheimer abnormally phosphorylated tau by dephosphorylation with protein phosphatase-2A, -2B and -1. Mol. Brain Res. 38, 200–208. 10.1016/0169-328x(95)00316-k 8793108

[B55] Wang J. Z. Grundke-Iqbal I. Iqbal K. (1996b). Glycosylation of microtubule-associated protein tau: an abnormal posttranslational modification in Alzheimer’s disease. Nat. Med. 2, 871–875. 10.1038/nm0896-871 8705855

[B56] Wang J. Z. Grundke-Iqbal I. Iqbal K. (2007). Kinases and phosphatases and tau sites involved in Alzheimer neurofibrillary degeneration. Eur. J. Neurosci. 25, 59–68. 10.1111/j.1460-9568.2006.05226.x 17241267 PMC3191918

[B57] Wang J. Z. Liu F. (2008). Microtubule-associated protein tau in development, degeneration and protection of neurons. Prog. Neurobiol. 85, 148–175. 10.1016/j.pneurobio.2008.03.002 18448228

[B59] Wang Y. Mandelkow E. (2016). Tau in physiology and pathology. Nat. Rev. Neurosci. 17, 5–21. 10.1038/nrn.2015.1 26631930

[B61] Wischik C. M. Novak M. Thøgersen H. C. Edwards P. C. Runswick M. J. Jakes R.. (1988). Isolation of a fragment of tau derived from the core of the paired helical filament of Alzheimer disease. Proc. Natl. Acad. Sci. U S A 85, 4506–4510. 10.1073/pnas.85.12.4506 3132715 PMC280459

[B62] Yan S. D. Chen X. Schmidt A. M. Brett J. Godman G. Zou Y. S.. (1994). Glycated tau protein in Alzheimer disease: a mechanism for induction of oxidant stress. Proc. Natl. Acad. Sci. U S A 91, 7787–7791. 10.1073/pnas.91.16.7787 8052661 PMC44487

[B63] Yin H. Kuret J. (2006). C-terminal truncation modulates both nucleation and extension phases of tau fibrillization. FEBS Lett. 580, 211–215. 10.1016/j.febslet.2005.11.077 16364303

[B64] Zhang Z. Song M. Liu X. Kang S. S. Kwon I. S. Duong D. M.. (2014). Cleavage of tau by asparagine endopeptidase mediates the neurofibrillary pathology in Alzheimer’s disease. Nat. Med. 20, 1254–1262. 10.1038/nm.3700 25326800 PMC4224595

[B65] Zilka N. Filipcik P. Koson P. Fialova L. Skrabana R. Zilkova M.. (2006). Truncated tau from sporadic Alzheimer’s disease suffices to drive neurofibrillary degeneration *in vivo*. FEBS Lett. 580, 3582–3588. 10.1016/j.febslet.2006.05.029 16753151

